# Aromaticity and Chirality: New Facets of Old Concepts

**DOI:** 10.3390/molecules29225394

**Published:** 2024-11-15

**Authors:** Bagrat A. Shainyan

**Affiliations:** Institute of Chemistry, Siberian Division of the Russian Academy of Sciences, 1 Favorsky Street, 664033 Irkutsk, Russia; bagrat@irioch.irk.ru

**Keywords:** aromaticity, electron-counting rules, chirality, dimensionality of space, boundaries of the Periodic Table of the Elements

## Abstract

The review summarizes the results of previous and the latest studies on aromaticity and related concepts. The electron counting rule for 3D-aromatic systems 2(n + 1)^2^ is shown to be a generalization of the 4n + 2 rule for planar molecules, and, vice versa, the latter can be derived from the former. The relative stability of the push–pull and captodative aromatic systems is shown to depend on the nature of the groups separated by the C=C bond in geminal or vicinal positions. The fully symmetrical molecules of hexamethylbenzene and hexacyanobenzene were studied using structural, energetic, and NMR criteria, and the donor substituents were shown to increase the aromaticity. Taking into account the coincidence of the number of π-electrons in aromatic systems with the number of electrons on the filled electron subshells (s, p, d, and f) and considering electrons as objects in a space of states allowed to conclude that no g-elements can exist and that the extension of the Periodic Table is possible only by filling 6f, 7d, or 8s subshells. The dimensionality of space also affects the chirality of molecules, making planar or even linear molecules chiral on oriented surfaces, which can be used for the preparation of enantiomerically pure drugs, resolution of prochiral compounds, etc.

## 1. Introduction

Considering the relationship between the properties of chemical objects and dimensionality of space they exist in is a challenging task. However, it is worth discussing, because it gives us a better understanding of how the dimensionality of space is connected with basic concepts such as aromaticity, chirality, and even the boundaries of the Mendeleev Periodic Table of Elements.

The concept of aromaticity is one of the most known and, at the same time, still remains one of the most intriguing in organic chemistry. First formulated as early as a century and a half ago by *A. Kekulé* [1], it was theoretically substantiated much later by *E. Hückel* [2]. Being initially limited to organic molecules, it was applied to planar conjugated cyclic compounds, including heterocycles and charged species. Later on, it was extended to inorganic compounds showing the so-called all-metal aromaticity [3,4,5]. Among the new types of aromaticity that have appeared in recent years are tubular (cylindrical) aromaticity in boron toroids [6,7], disk aromaticity in the B_30_ boron cluster of a bowl structure [8,9,10], and cubic aromaticity in polynuclear zinc clusters [11]. The latter types of aromaticity refer to organometallic spatial rather than planar structures. Three-dimensional aromaticity is also intrinsic to some types of organic compounds, such as fullerenes [12], some cage structures (cubane, adamantine [13], etc. [14]), closo-carboranes [15], or cyclobutadiene dianion salts [16,17], although, as recently stated by *M. Solà* et al. [18], the latter structures should be considered as “2D-aromatic-in-3D” but not truly 3D-aromatic. A relationship between the 2D and 3D π-aromaticity was established for a macrocyclic cage in which two aromatic porphyrin units are bridged by four thiophene arms [19]. As argued in [20,21], two types of 3D aromaticity should be distinguished: spherical aromaticity, applicable, e.g., to fullerenes, for which rolling out the sphere over the surface does not destroy the aromaticity completely, and spatial aromaticity applicable to cage structures, for which the breaking of any bond of the cage fully destroys the aromaticity of the whole molecule. Note that, recently, *M. Solà* et al. showed that even strong structural changes of closo-carboranes by going to their *nido* counterparts do not destroy (although reduce) the aromaticity [22]. The group theory method was applied to explain and unite different electron count rules (*Hückel*, *Baird*, *Möbius*, etc.) [23].

In an “opinion article” [24], *M. Solà* considered the existence of so many specific types of aromaticity as one of four reasons (along with too many descriptors, multidimensional character, and unwarranted use) for the low reputation of the aromaticity concept. I would not say “low reputation”; rather, it is a multifaceted concept that still attracts the ongoing interest of the researchers. It is evidenced by recent publications, in which the perspectives on this fundamental idea are outlined by posing and answering key questions about the essence of aromaticity [25], or by the review stating that aromaticity is a theoretical notion, so it is theory-dependent [26].

## 2. Results and Discussion

### 2.1. Push–Pull Versus Captodative Aromaticity

Of special interest for the present review is the push–pull and captodative aromaticity, which was first proposed by us [27] in 2008 as an extension of the studies of [*n*,*m*]-fulvalenes (*n*,*m* = 3, 5, 7) [28] to their vinylogs.

**Figure 1:**
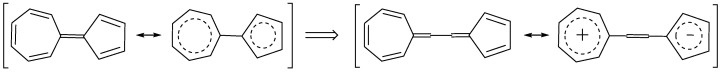
Transition from fulvalenes to their vinylogs on the example of pentaheptafulvalene.

**Figure 2:**
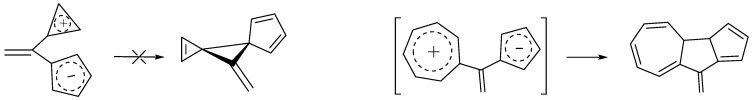
Stable mesoionic geminal vinylog of calicene and its unstable tropylium analog.

The last structure in Figure 1 represents what we called the “push–pull” aromaticity with concerted unidirectional transfer of the electron density from one cyclopolyenyl ring to another. However, one can imagine a mesoionic isomer of this structure, with the two cyclopolyenyl rings attached to the same carbon atom of the C=C double bond, for which the principal question is whether it corresponds to a minimum on the potential energy surface (PES) or “rolls down” to the minimum corresponding to the covalent structure without charge separation? The answer turned out to depend on the nature (size) of the cyclopolyenyl rings. Thus, the mesoionic geminal vinylog of calicene with the cyclopropenyl and cyclopentadienyl rings is a minimum on the PES, as well as its covalent isomer, 9-methylenedispiro[2.0.4.1]nona-1,5,7-triene, whereas its analog with the tropylium ring is optimized to 9-methylidene-3b,9-dihydro-3a*H*-cyclopenta[*a*]azulene (Figure 2) [29]. Apparently, the observed different behavior is due to steric hindrances appearing upon cyclization of the former mesoionic compound and their absence in the case of the latter. In particular, the rapprochement of the carbon atoms adjacent to the carbons directly attached to the double bond with retention of planarity is possible for the tropylium derivative but not for the cyclopropenylium one.

Note that mesoionic vinylog of calicene is a local minimum lying 2.95 kcal/mol above the product of its possible cyclization in the gas phase [29]. However, the product of cyclization without charge separation is much less polar than the mesoionic structure, which, due to a much larger dipole moment, becomes 2.82 kcal/mol more stable even in chloroform and 5.25 kcal/mol more stable in DMSO [29].

At first glance, the push–pull structures, in which the donor and acceptor groups at different carbons of the C=C bond act concertedly, should be more stable than their captodative isomers with the same groups in the geminal position, opposing each other in polarization of the C=C bond to which they are attached. This very result was obtained in our earlier work on the push–pull and captodative aromatic systems with the cyclopropenyl and cyclopentadienyl groups, where a large energy difference of 38.0 kcal/mol in favor of the push–pull structure was obtained [27]. No other groups at the C=C bond have been studied, so the most interesting question remained open: whether it is possible to find such groups at the C=C bond that would make the non-fully conjugated captodative structures more stable than their fully conjugated push–pull isomers. No such question was posed so far. In a search for an answer, the push–pull and captodative aromatic systems with the tropylium cationic and cyclopentadienylium motifs at one carbon atom of the C=C bond and a variety of electron-donating or -withdrawing groups at the other or the same carbon atom have been analyzed in the recent works using different (NMR, structural, and energetic) criteria of aromaticity [29,30].

**Figure 3:**
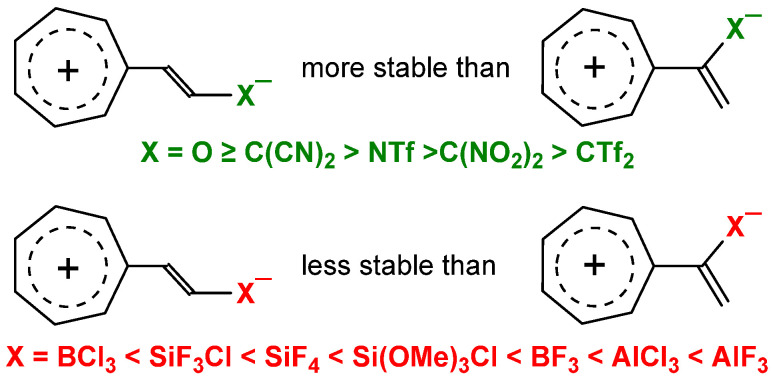
Different order of stability for organic and organometallic electronoacceptor groups X in the push–pull and captodative tropylium derivatives.

To summarize briefly, the answer is “yes”; as shown in Figure 3, for typical organic electron acceptor groups X = O, TfN, (NC)_2_C, (NO_2_)_2_C, and Tf_2_C, the push–pull tropylidene derivatives [tropylium]^+^–CH=CH–X^−^ are expectedly more stable than their captodative isomers [tropylium]^+^C(X^−^)C=CH_2_, with the Δ*E* dropping in this order from 32.6 to 15.7 kcal/mol (in gas) or from 23.9 to 7.50 kcal/mol (in solution) [29], the lowest Δ*E* values being obtained for the strongest acceptor Tf_2_C. In contrast, for organoelement or metal-containing groups, the order of stability is inverted, with the energy preference of the captodative isomer reaching 9.97 (in gas) or 1.91 kcal/mol (strongly attenuated in solution) for X = AlF_3_ [29].

The higher aromaticity of the captodative isomers for organoelement or metal-containing X groups is manifested not only in their higher stability but is also proven by magnetic and structural criteria [29]. Even when they are less stable, the captodative structures may possess a higher aromaticity according to the magnetic (NICS) and structural (equalization of the C-C bonds in the ring) criteria, because being mesoionic compounds, they cannot be drawn without charge separation.

This remarkable and unexpected result was explained assuming that, in the push–pull isomers, the electron density is transferred to the π-system of the substituent, while, in the captodative isomers, it can be accepted only to the σ*(C–X) orbital. Thus, when the electronodonor and electronoacceptor effects act independently, through the σ or π system, the non-fully conjugated captodative isomers can be more stable and more aromatic than their push–pull isomers with a more extended conjugation system.

**Figure 4:**
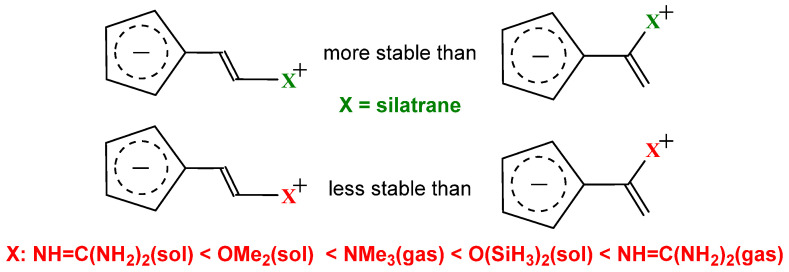
The order of stability for the push–pull and captodative cyclopentadienylium anionic derivatives as a function of substituent X and the medium.

Next question is how general is the conclusion that the nature of the substituent may affect the relative stability of the push–pull and captodative isomers; in particular, does it depend on the sign of the charge on the aromatic ring? This problem was examined by calculating cyclopentadienyl derivatives possessing an aromatic cyclopentadienyl ring and organic electronoreleasing groups such as NR_3_, R_2_O, (NH_2_)_2_C=NH, O(SiH_3_)_2_, and silatrane. As follows from Figure 4, for typical organic π-donors (X = N, O), the captodative isomers of the cyclopentadienylium anionic structures are more stable; noteworthy here is the dependence of the relative stability on the medium: for the guanidinium derivatives, the energetic preference is minimal in solution (4.77 kcal/mol) and maximal in gas (12.9 kcal/mol) in the whole series of the studied compounds [30].

An interesting question is how does the polarization of the C=C bond in the two types of isomers depend on the solvent and the substituent? The direction of polarization in the push–pull derivatives is evident: to the ring in the tropylium species or to group X in cyclopentadienylium ones. For the former species with “conventional” organic substituents X, the polarization increases in going from gas to solution, whereas, for those with organoelement substituents X, it decreases up to the inversion. The push–pull cyclopentadienylium derivatives show a similar behavior [30].

In the captodative derivatives, the polarization varies irregularly, depending on both X and the medium (see [30] for details). Surprisingly, in spite of being polarized towards the internal carbon atom (that is, in the direction to the ring), the C=C bond in the tropylium derivatives containing organoelement group X is electron-rich both in gas and in solution. That means that the π-electron density donation from the C=C bond to the aromatic ring is outweighed by the σ-donor effect of X to the C=C bond. Following the same logic, the C=C bond in most of captodative cyclopentadienyl species is polarized in the direction from the ring towards the terminal carbon and is electron-deficient.

### 2.2. Electron Counting Rules

Inalienable from the problem of aromaticity are the electron counting rules. The first and the best known is the *Hückel* 4n + 2 rule [2] originally proposed for planar cyclic molecules with a closed system of conjugation. Much later, the *Hirsch* 2(n + 1)^2^ rule was proposed for fullerenes and other cage-like systems possessing three-dimensional aromaticity [12,31,32,33,34]. For particular classes of organic compounds, other rules were proposed, like the Wade-Mingos rules for polyhedra such as closoboranes [35,36] or the jellium superatom model for post-transition element clusters [37] (for the comparison of the two, see [37]). Note also that the *Hirsch* 2(n + 1)^2^ rule was generalized to the open-shell spherical aromatic systems in the form of the (2n^2^ +  2n  +  1) rule [38].

When considering the *Hückel* and the *Hirsch* rules, the questions arise: what is so special about these formulae, why are they expressed as they are, and how are they connected to each other (if at all)? The only thing that can be said a priori about the numbers of electrons in these two formulae, regardless of specific objects they describe, is that both are even; however, 4n + 2 is not just even but twice the odd number, whereas, for 2(n + 1)^2^, it is not always the case. In an attempt to find an answer, we considered the number of irreducible states in an arbitrary n-dimensional space, abstracting from the specific nature of the objects in it; in other words, in the space of states. A zero-dimensional space has only one state—that is, a point. A one-dimensional space has three irreducible states, namely, a point, a positive vector, and a negative vector. The same logic gives us five and seven irreducible states in the 2D and 3D spaces, respectively (in the latter case, these are “point”, “to the left”, “to the right”, “up”, “down”, “forward”, and “backward”). These options exhaust the possibilities of our three-dimensional space.

Note that there is an important difference between the physical space and the space of states. While the physical space we live in is three-dimensional, the space of states is considered as a set of irreducible states in an arbitrary n-dimensional space ignoring the nature of the objects in it. The number of irreducible states in an n-dimensional space of states is (2n + 1). Considering electrons as the objects, the space of states becomes a four-dimensional manifold due to four possible shapes of electron orbitals: s, p, d, and f. While s orbitals are non-directional, p orbitals have a dumbbell shape, d orbitals are cloverleaf or torus shapes, and f orbitals are a tetrahedral shape.

If to return to the electron counting rules and consider electrons as the objects in this space, the *Pauli* principle is fulfilled (maximum two electrons in each state), and the number of electrons in the *n*-dimensional space of states is 2 × (2n + 1) = 4n + 2, exactly coinciding with the *Hückel* rule. If to take into account that any *n*-dimensional space includes all the (n − k)-dimensional subspaces, the total number of electrons is given as the sum over all k ≤ n, and, according to the formula,

$$\sum_{k=0}^{n}a_{k}=\frac{\left(n+1\right)}{2}(a_{o}+a_{n}),$$

is 2(n + 1)^2^:

$$\sum_{k=0}^{n}[4(n-k)+2]=\frac{\left(n+1\right)}{2}(4n+2+2)={2(n+1)}^{2}$$

exactly coinciding with the *Hirsch* rule.

Therefore, we get the *Hirsch* 2(n + 1)^2^ rule as a generalization of the *Hückel* 4n + 2 rule by summing over all possible (n − k)-dimensional subspaces embedded into an *n*-dimensional space of states. In other words, as a two-dimensional space is a part of a three-dimensional space, the *Hückel* rule for planar aromaticity is a particular case of the more general *Hirsch* rule for spherical aromaticity. Noteworthy, for even n, the number of electrons by the *Hirsch* 2(n + 1)^2^ rule coincides with the number of electrons by the *Hückel* (4m + 2) rule for m = ½n(n + 2).

Remarkably, not only the *Hirsch* rule can be derived from the *Hückel* rule, but, vice versa, the *Hückel* rule can also be derived from the *Hirsch* rule by calculating the difference Δ between the number of electrons for two sequential systems obeying the *Hirsch* rule and having, respectively, n and (n − 1) electrons.

Δ = 2(n + 1)^2^ − 2[(n + 1) − 1]^2^ = 4n + 2

In other words, as the number of objects in the *n*-dimensional space is determined by summing their number over all included (n − 1)-dimensional subspaces, their number in the (n − 1)-dimensional subspace is determined by subtracting the number of objects in the n-dimensional space from that in the (n + 1)-dimensional space.

In [24], *P. Kumar* showed how the use of orthogonal SO(2) or SO(3) groups are connected, respectively, with the *Hückel* 4n + 2 and *Hirsch* 2(n + 1)^2^ electron counting rules, whereas the infinite dihedral D_∞_ group is connected with the *Baird* 4N rule.

Interestingly, the relationship between the two rules dates back to the early work of *Rubin and Ordóñez* [39], who constructed symmetric tensor harmonics of arbitrary rank on ≥3D spheres and determined the degeneracy of eigenvectors (orbitals) in the 2D space as (2*l* + 1) and that in the 3D space as (*l* + 1)^2^.

One can wonder whether all planar aromatic systems obey the *Hückel* rule and all systems possessing 3D aromaticity obey the *Hirsch* rule. Strictly speaking, they do not. However, the 4n + 2 rule has no exceptions provided it is correctly used. The cases of apparent violation of the *Hückel* rule in aromatic polycyclic systems with 4n π-electrons (acenaphthylene with 12 π-electrons, pyrene with 16 π-electrons, and coronene and isocoronene with 24 π-electrons [40]) mean that the rule must be applied not to all but only to the peripheral π-electrons forming the ring current.

More complicated is the situation with the *Hirsch* 2(n + 1)^2^ rule. As was assumed in [20,21], two types of 3D aromaticity can be distinguished. The first one, defined as “spherical”, excellently describes the aromaticity of fullerenes and heterofullerenes [31,32,34,41,42,43], although, as was remarked, it is meaningless to discuss such structures as C_60_^10+^ or C_60_^12−^, which, according to the 2(n + 1)^2^ rule, are aromatic [44].

*Reiher* and *Hirsch* connected the electronic structure of spherically symmetric molecules with the electronic structure of the inert gas-filled shell by a mental experiment—the increase of the atom from a point to a hollow sphere with uniform positive charge distribution over the surface and further to the corresponding polyhedron with violation of the uniform distribution of the charge and its localization in the vertices of the polyhedron [45]. Interestingly, with the increase in the radius of the sphere (pseudoatom), the order of the energy levels varies from “normal” (1s, 2s, 2p, 3s, 3p, 3d, 4s, 4p, …) to the “inverted” (1s, 2p, 3d, 2s, 3p, 3s, 4p, 4s, …) due to stronger destabilization of the s-levels (initially stronger bound) with removal from the core. The authors also discussed the limits of applicability of the 2(n + 1)^2^ rule, which is fulfilled, because the level orbital energies become closer to the size of the spherical structure and the corresponding states are mixed.

The second type, which we called “spatial” [20,21], is applicable to various cage compounds [13,16,17,46]. The difference between the “spherical” and “spatial” aromaticity is that destroying, for example, the fullerene sphere by unfolding it onto a plane does not destroy the aromaticity completely (it is retained in the plane). In contrast, breaking any bond in a cage structure converts it to a monocyclic structure and fully destroys the aromaticity. Therefore, the conclusion can be made that the *Hirsch* rule is primarily applicable to the systems with “spherical” (like in fullerenes) or “cylindrical” aromaticity [6,42], whereas the *Hückel* rule – to the systems with “spatial” aromaticity [13,16,17,35,46,47,48,49], etc.

The *Hückel* rule determines the number of π-electrons in the aromatic systems (2, 6, 10, or 14), which surprisingly (but hardly accidentally) coincides with the number of electrons on the filled electron subshells of atoms (s, p, d, and f). In contrast, the number of electrons defined by the *Hirsch* rule corresponds to that on the filled electron shell of an inert gas (2, 8, 18, 32,…) and differs from that on the filled subshells of atoms (although, sometimes, the two may coincide).

In conclusion, it should be stressed that both the planar and three-dimensional aromaticity, as well as the corresponding electron counting rules, have a common origin. It is related, on the one hand, to the formal consideration of an arbitrary *n*-dimensional space of states and, on the other hand, to the fact that all elementary particles in chemistry (electrons, protons, and neutrons), as the objects in this space, are fermions, obeying the *Fermi-Dirac* statistics and, consequently, the *Pauli* exclusion principle.

**Figure 5:**
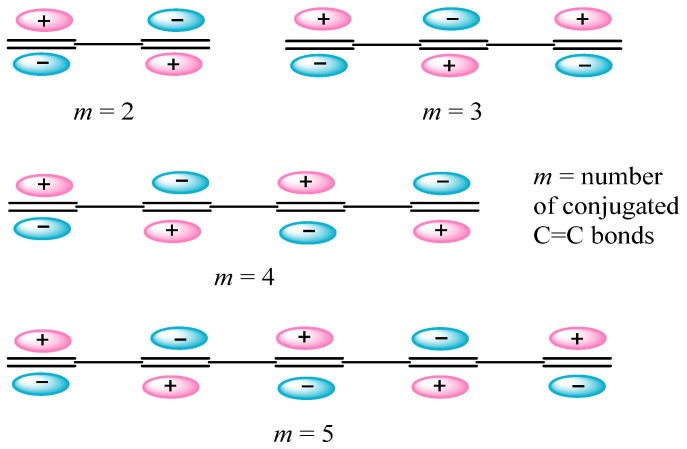
Nodes between the alternating single and double bonds in the HOMOs of polyenes.

The aforementioned even number of electrons predicted by the *Hückel* and *Hirsch* rules is due to doubling according to the *Pauli’s* principle. But why it is an odd number of electrons that is doubled in the *Hückel* rule? The answer becomes clear from considering the nodal properties of the highest molecular orbitals (HOMOs) of linear conjugated polyenes (Figure 5). The carbon chain of alternating ordinary and double bonds in conjugated dienes can be closed to the Hückel aromatic hydrocarbon in the binding mode only by overlapping the HOMO lobes of the same sign at the terminal C=C bonds, which is the case only if the number of the conjugated terminal C=C bonds *m* is odd.

The simplest example is the transformation of the HOMO of 1,3,5-hexatriene with two nodes (π3) into the HOMO of benzene with one nodal plane (π3) passing through the opposite carbon atoms upon cyclization (This is true only for cyclization to the *Hückel* hydrocarbons; we are not concerned with the *Möbius* aromaticity in [4n]annulenes here (for review, see [50])). In fullerene C_60_ obeying the *Hirsch* rule, the nodal element is a sphere in which all carbon atoms lie [51], the HOMO lobes of the opposite sign being directed inside and outside this sphere.

### 2.3. Effect of the Electron Density on the Aromatic Ring

Another non-obvious issue with aromaticity is its dependence on the electron density on the aromatic ring. Intuitively, the more the electron density, the higher the aromaticity. However, there are only a few works that have shown a definite answer to this question. Thus, the fluorination of benzenes decreases the aromaticity due to the overall electronoacceptor effect of the fluorine atoms [52]. It has been declared that both electron-releasing and electron-withdrawing groups decrease the aromaticity of the ring due to the partial localization of p-electrons [53,54]. The so-called *para*-delocalization index (PDI) has been stated to correlate nicely with substituent constants (higher PDI reflecting greater aromaticity) [53], although there was only one strong acceptor substituent (NN^+^) that extended the range of constants to a diapason suitable for acceptable correlation. The effect of a strong electronodonor (silatranylmethyl group) on the aromaticity of N-heterocycles was studied using structural and energetic criteria [55], and a conclusion was that a strong electron-donating silatranyl group increases the aromaticity.

To avoid the problems connected with violation of the symmetry of the aromatic ring (such as the aforementioned localization of p-electrons), we calculated and compared two fully symmetrical molecules, hexamethylbenzene and hexacyanobenzene, using structural, energetic, and nuclear magnetic criteria (All calculations were performed at the MP2/cc-pVTZ level of theory using the Gaussian09 program package. The NICS values were calculated within the GIAO approximation).

Structurally, the molecules are very similar, as the only difference is the slightly longer C–C ring bonds for X = CN (1.4053 Å) than for X = Me (1.4042 Å). The molecule of hexacyanobenzene is somewhat looser when judged from the distance between the *para* carbon atoms: 2.8106 Å for X = CN and 2.8081 Å for X = Me), although the difference is too small to be convincing.

The NICS(0), NICS(1), and NICS(2) values for the two molecules were calculated at the distances from the center of the benzene ring of 0.0, 1.0, and 2.0 Å, respectively, and are given in Table 1.

Negative NICS values in the center of the ring or above it imply the presence of an induced diatropic ring current indicating aromaticity; the more negative the NICS, the higher aromatic the molecule.

As per the NICS values, hexamethylbenzene is more aromatic than hexacyanobenzene in the plane of the molecule, although, as a nucleus is removed from the center, the difference decreases at 1.0 Å, and at 2.0 Å, there is a reversal of the aromaticity. This may be due to the anisotropic contribution from six cyano groups above the plane of the ring. Apparently, the NICS values in the center of the benzene ring are more appropriate as a criterion of aromaticity. The higher aromaticity of hexamethylbenzene is consistent with the results obtained in [52] for benzene and its various fluorosubstituted derivatives.

Even more persuasive is the energetic criterion, which was estimated by calculating the aromatic stabilization energy (ASE) as the energetic effect of the following homo-desmotic reaction and given in Table 1.

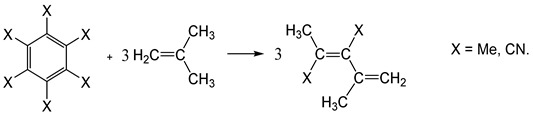



The difference between the ASE values is very large: 51.80 kcal/mol (X = Me) and 3.97 kcal/mol (X = CN), indicating a much higher aromaticity of hexamethylbenzene and proving the strengthening effect of electronodonor substituents on the aromaticity.

### 2.4. Aromaticity and the Boundaries of the Periodic Table of the Elements: What Is the Connection?

The above simple formal approach based on considering electrons in an arbitrary space of states surprisingly leads to another unexpected conclusion concerning the boundaries of the Periodic Table of Elements, the problem that still bothers the scientific community. The last discovered chemical element is Oganesson ^118^[Og], named after Yuri Oganessian. It is the heaviest noble gas that completes the seventh period of elements and has the electron configuration [Rn]5f^14^6d^10^7s^2^p^6^ or 1s^2^2s^2^p^6^3s^2^p^6^d^10^4s^2^p^6^d^10^f^14^5s^2^p^6^d^10^f^14^6s^2^p^6^d^10^7s^2^p^6^. If the number of possible types of subshells is, indeed, related to the number of possible states in our three-dimensional space (“point”, “to the left”, “to the right”, “up”, “down”, “forward”, and “backward”), the s, p, d, and f types of orbitals exhaust the possible types of the electron levels. Hence, no g-elements (to say nothing of h-elements) can exist. It does not mean that the Periodic Table of Elements cannot be extended beyond the seventh period but just that the first so-called g-element Unbiunium, ^121^[Ubu] (eka-actinium), may have the [Og]8s^2^6f^1^, [Og]8s^2^7d^1^, or [Og]8s^2^p^1^ configuration rather than [Og]8s^2^5g^1^, as predicted earlier (http://en.wikipedia.org/wiki/, accessed on 15 September 2024). This is supported by the fact that the application of the relativistic-coupled cluster (RCC) method shows for eka-actinium the [Og]8s^2^p^1^ configuration as the ground state, which is 0.4 eV lower in energy than the lowest [Og]8s^2^7d^1^ configuration (see [56] and the references cited therein).

### 2.5. Chirality and Dimensionality of Space

**Figure 6:**
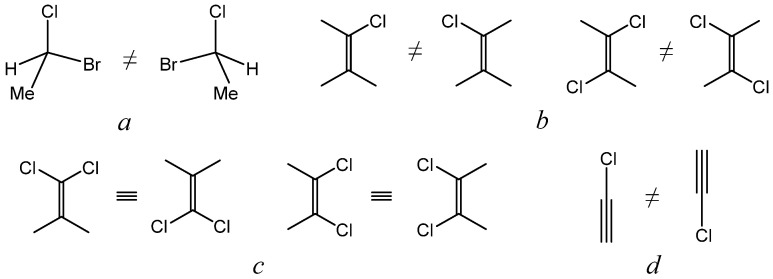
Equivalence and non-equivalence of simple molecules in three-dimensional (**a**), two-dimensional (**b**,**c**), and one-dimensional space (**d**).

Not less interesting and having an intriguing relationship with the dimensionality of the space is another chemical notion, the chirality, which is the property of a molecule that does not coincide with its mirror image. The dependence of chirality on the dimensionality of space is presented in Figure 6. For example, a chiral molecule of 1-bromo-1-chloroethane does not superimpose with its mirror image by translation or rotation in three-dimensional space (Figure 6a), so the compound exists as a pair of enantiomers. Less common are “chiral” molecules in a space of lower dimensionality. In a two-dimensional space, in the plane of the paper (Figure 6b), vinyl chloride and *trans*-1,2-dichloroethylene would also exist in two “enantiomeric” forms, because their mirror images can be superimposed only by out-of-plane rotation, which is forbidden in a two-dimensional space. In contrast, the mirror images of the molecules of *cis*-1,2-dichloroethylene or vinylidene chloride can be superimposed by the allowed 180° in-plane rotation (Figure 6c). Finally, in one-dimensional space, “enantiomers” would exist even for the molecule of chloroacetylene and similar linear structures, because the two forms cannot coincide by translation along the axis of the molecule, which is the only allowed type of movement in one-dimensional space (Figure 6d) [57].

Once in a chiral surrounding, planar or even linear molecules like those in Figure 6b,d may become distinguishable due to different interactions with an oriented surface. The fact that the equivalence or non-equivalence of the molecules in Figure 6b–d is not a mind game is indirectly supported by studying the possibility of the existence of space with non-integer dimensionality. Thus, as early as in 1985, the comparison of the experiment and theory gave the dimensionality of the Minkowski spacetime as 5.3 × 10^−7^ less than 4 [58]. Recently, using an information theoretical approach, the conclusion was made that the dimensionality of a space can be non-integer; in particular, it was mathematically justified that some systems in our real physical world are better described by non-integer dimensionality—in particular, ~2.718 (the base of natural logarithms)—rather than 3 [59]. Moreover, it was declared that non-integer dimensions might be useful and may have measurable effects.

Experimentally, the non-equivalence of some achiral and prochiral molecules was proven by enantiospecific chemisorption [60] or specific adsorption on the oriented (chiral) surfaces [61,62] and the use of chiral surfaces for enantioselective processes [63,64,65]. The chirality of the surface/adsorbate system can be achieved by the adsorption of a chiral adsorbate [66] on an achiral surface or by the adsorption of a prochiral adsorbate on a chiral surface [67]. Chiral surfaces can be used for the preparation of enantiomerically pure drugs [63] and have other practical applications, such as for the kinetic resolution of prochiral compounds by adsorption/desorption on an oriented (chiral) surface.

## 3. Conclusions

The problems of how the dimensionality of space is connected with basic chemical concepts such as aromaticity and chirality, how the different electron counting rules applicable to different types of aromaticity are interrelated, and what is common between the number of π-electrons in aromatic systems and the boundaries of the Periodic Table of Elements are discussed. In addition to planar aromatic molecules, numerous different types of 3D aromatic systems have appeared, which aromaticity should be divided into “spherical” and “spatial”. The *Hückel* 4n + 2 electron counting rule for planar molecules and the *Hirsch* 2(n + 1)^2^ rule for cage-like 3D-aromatic systems are shown to be interrelated: the *Hirsch* rule is a generalization of the *Hückel* rule, and, in turn, the *Hückel* rule can be derived from the *Hirsch* rule.

Considering electrons as the objects in an arbitrary space of states and taking into account the coincidence of the number of π-electrons in aromatic systems with that on the filled s-, p-, d-, and f-subshells allowed to conclude that no g-elements can exist and that the extension of the Periodic Table of Elements is possible only by the filling of 6f, 7d, or 8s subshells.

Investigation of the effect of the substituent on the aromaticity for symmetrical molecules, C_6_Me_6_ and C_6_(CN)_6_, using various criteria of aromaticity showed that, while, structurally, the two molecules are very similar, the NICS values are indicative of a higher aromaticity of the former as compared to that of the latter, and the aromatic stabilization energies unequivocally prove the much larger aromaticity of C_6_Me_6_, which has the ASE of 51.80 kcal/mol, versus only 3.97 kcal/mol for C_6_(CN)_6_.

Detailed analysis of the relative stability of the push–pull and captodative aromatic systems with the donor and acceptor moieties separated by the C=C bond in a geminal or vicinal manner revealed that, as distinct from the earlier study, the captodative isomers can be more stable than their push–pull counterparts in spite of concerted (unidirectional) electronic effects of the donor and acceptor groups in the latter. This unusual effect was explained by the independent interaction of the BHlg_3_, AlHlg_3_, and similar groups with the σ- and π-electronic systems of the C=C bond.

The dimensionality of space critically affects the chirality of molecules, making planar or even linear molecules chiral on oriented chiral surfaces. This effect can be used for the synthesis of enantiomerically pure drugs or the kinetic resolution of prochiral compounds.

Finally, it should be noted that the above analysis does not lay claim to be exhaustive, impeccable, or infallible, but rather, its main goal is to encourage further discussion and to provoke criticism rather than to avoid it.

## Figures and Tables

**Table 1:** Nucleus-Independent Chemical Shifts in and above the ring center, and aromatic stabilization energies of the molecules of hexamethyl- and hexacyanobenzene.

Molecule	–NICS			ASE
	0.0 Å	1.0 Å	2.0 Å	
Hexamethylbenzene	12.19	10.98	5.13	51.80
Hexacyanobenzene	9.00	10.57	5.34	3.97

## Data Availability

Not applicable.
